# Survey on general practitioners’ and pharmacists’ opinions regarding patient-initiated treatment of recurring urinary tract infections

**DOI:** 10.1007/s11096-021-01295-2

**Published:** 2021-06-10

**Authors:** Rian Lelie- van der Zande, Ellen S. Koster, Martina Teichert, Marcel L. Bouvy

**Affiliations:** 1 Department of Pharmacoepidemiology and Clinical Pharmacology, Utrecht Institute for Pharmaceutical Sciences, Utrecht University, Utrecht, The Netherlands; 2 KNMP (Royal Dutch Pharmacists Association), The Hague, The Netherlands; 3 Department of Clinical Pharmacy & Toxicology, Leiden University Medical Center, Leiden, The Netherlands

**Keywords:** Anti-bacterial agents, Attitude, General practitioners, Pharmacists, Treatment, Urinary tract infections

## Abstract

*Background* The Dutch general practitioners (GP) guideline for urinary tract infections (UTI) recommends patient-initiated treatment for women with recurring UTI. In countries other than the Netherlands, community pharmacists play a role in dispensing antibiotics for recurring UTI without preceding GP consultation. *Objective* To study GP and pharmacist opinions regarding the desirability of patient-initiated treatment, including potential pharmacist support for, and consequences of, facilitated access to antibiotics. *Setting* Dutch community pharmacies that cooperate with at least two GPs in their regional primary care network. *Method* Pharmacists in a postgraduate education program invited their residency pharmacist and 2–3 GPs to anonymously complete an online questionnaire. Questions related to diagnosis, treatment and potential role of the pharmacist. Answers were formulated as multiple-choice or ratings on a 5-point Likert scale. Data were analysed per professional group using descriptive statistics. Answers of pharmacists and GP to corresponding questions were analysed using a Chi-square test (*p* < 0.05). *Main outcome measure* Desirability of patient-initiated treatment and supporting role of the pharmacist. *Results* A total of 170 GPs and 76 pharmacists completed the questionnaires. Of the GPs, 35.1% supported patient-initiated treatment. Of the pharmacists, 69.7% were willing to dispense an antibiotic to a patient without preceding GP consultation after performing a probability check. In total, 65.7% of GPs and 44.7% of pharmacists thought that facilitated access to antibiotics would increase use of antibiotics (*p* < 0.05). *Conclusion* Support of GPs for facilitated access to antibiotic treatment by patient-initiated UTI treatment was limited, even with pharmacist support. The majority of pharmacists were willing to dispense an antibiotic after a probability check of an episode of recurring UTI, but both pharmacists and GPs were concerned about overuse of antibiotics.

## Impacts on practice

- Additional agreements should be made between general practitioners and pharmacists regarding how to support women with recurring UTI.
- Pharmacists and GPs should discuss GP concerns about patient-initiated treatment with pharmacist support.
- Pharmacists should follow an accredited training program prior to implementation of patient-initiated treatment in the pharmacy.
- Supporting materials (e.g., a checklist for verification of symptoms, materials for pharmacotherapeutic audit meetings) may help GPs and pharmacists implement services for women with recurring UTI.

## Introduction

Uncomplicated urinary tract infections (UTI) are among the most common acute conditions for women in primary care, with a lifetime prevalence of 40–60% [1, 2]. Most women experience their first UTI before age 25 [1]. A UTI is the most frequent indication for an antibiotic prescription in primary care in the Netherlands [3]. In most countries, women with recurring UTI require a prescription for an antibiotic.

The Dutch General Practitioners UTI guideline recommends that general practitioners (GPs) discuss the symptoms of recurring UTI and self-initiated treatment with their female patients. If patients recognize the symptoms, GPs can provide them with an antibiotic prescription to be filled "when needed" [4]. This patient-initiated treatment (PIT) is supported by a study finding that 84% of otherwise healthy women aged 18–51 years could accurately self-diagnose and self-treat recurring UTI [5].

To date, antibiotic use and bacterial resistance in the Netherlands are relatively low [6, 7]. These statistics may be attributable to relatively good adherence of GPs to the prescribing guidelines, which are reinforced by regular pharmacotherapeutic audit meetings between GPs and pharmacists [8]. As a prerequisite, facilitated access to UTI antibiotics for PIT should not increase bacterial resistance.

In many countries, treatment for chronic diseases is shifting from hospital to primary care, due to the aging of the general population and the increased length of time people live at home. As GPs and practice nurses face increasing workloads [9], pharmacist provision of treatment for minor ailments such as recurring UTI may relieve some of the burden for GPs and their practices. According to study findings, it is possible to shift appropriate care duties, such as a probability check of an episode of recurring UTI, from the GP to the community pharmacist [10].

In some Canadian provinces and in New Zealand, women with recurring UTI can consult a trained pharmacist to receive an antibiotic without a preceding physician prescription [10–12]. In the Netherlands, community pharmacists may advise patients on non-prescription medicines for minor ailments, but they are not allowed to dispense prescription-only medicines such as antibiotics without a physician prescription. However, community pharmacists in the Netherlands may refill prescriptions if the physician has explicitly stated the number of times a prescription may be refilled [13].

Pharmacist support for women who choose to self-initiate treatment for UTI may provide GPs with additional confidence in this process. The resulting support of GPs for self-initiated treatment could enable a growing number of women to choose this option.

### Aim of the study

The aim was to study the opinions of GPs and pharmacists regarding the desirability of patient-initiated treatment of recurring UTI. The potential role of the pharmacist in this process was studied, as were the potential consequences for antibiotic dispensing of facilitating access to antibiotics through patient-initiated treatment with pharmacist support.

### Ethics approval

This study was approved by the coordinator of the postgraduate workplace-based curriculum for community pharmacist specialists. Use of observational data in descriptive studies in the Netherlands is not considered to be an interventional trial, according to Directive 2001/20EC of Dutch legislation [14, 15]. Therefore, the study protocol did not need to be submitted to a medical ethics committee for approval.

## Method

### Study design and inclusion

A cross-sectional study was performed using an online survey for GPs and community pharmacists in the Netherlands. Data were collected between March and July 2018. Pharmacists from 76 pharmacies, all of whom were enrolled in a postgraduate education program to become community pharmacy specialists, recruited study participants. Each postgraduate trainee personally invited the residency pharmacist, and 2–3 general practitioners from practices in their regional primary care network to anonymously complete an online questionnaire.

### Questionnaire design

Separate questionnaires were designed for GPs and pharmacists. The questionnaires were developed based on the findings of semi-structured face-to-face interviews with 3 GPs and 3 community pharmacists about procedures in GP-practice and PIT. The main topics addressed in the GP questionnaire included the current practice regarding diagnosis and treatment of recurring UTI and the ability of women to self-initiate treatment. In the pharmacist questionnaire, the main topics addressed were the willingness to check the probability of recurring UTI and implementation needs. Both questionnaires were piloted by 2 pharmacists and 2 GPs, respectively, to evaluate clarity and presentation and identify missing items. The questionnaires included questions with predefined options (with or without open response boxes) and statements to be ranked on a 5-point Likert scale that ranged from “totally disagree” to “totally agree”.

### Data collection

The postgraduate trainees e-mailed the participating GPs and residency pharmacists a link to the questionnaire with a research code for identification of the questionnaire. The researchers had access to the submitted anonymized responses. Personal data were accessible to the postgraduate trainee only. The researchers communicated with the postgraduate trainees through the coordinator of the curriculum, who acted as a ‘trusted third person’. If needed, the GPs and supervising pharmacists received two reminders from the trainees to complete the questionnaire. The survey was conducted using an online survey system (Questback Version 27).

### Data analysis

Descriptive statistics were calculated, and answers were analysed for each professional group. For the Likert scale questions, a positive answer was calculated by adding “agree” and “totally agree” responses, a negative answer was calculated by adding “not agree” and “totally not agree” responses. Answers of pharmacists and GPs to corresponding questions were analysed using a Chi-square test (*p* < 0.05). All answers were analysed according to the gender and age of the respondents using a Chi-square test (*p* < 0.05). A cut-off of 45 years (median age of GPs and pharmacists) was used for age. Open responses were coded and counted and percentages were calculated. All data were analysed using IBM SPSS for Windows, version 23.0.

## Results

### Response

The questionnaires were completed by 76 community pharmacists and 170 GPs from practices cooperating with the pharmacies. Of all invited GPs, 25 did not complete the questionnaire. Table 1 shows the characteristics of GPs and pharmacists.

**Table 1:** Characteristics of community pharmacists and GPs

	Community pharmacists	GPs
Total number of participants (% of all pharmacists/GPs working in pharmacy/GP practice in 2018)	76 (3.8%)	170 (2.2%)
Number of female participants (% of all participants)	37 (48.7%)	92 (54.1%)
Mean age (in years ± sd)	45.7 ± 9.8	46.0 ± 10.5
Number of participants 45 years or older	32 (42.1%)	77 (45.3%)

### Opinions of GPs and pharmacists regarding self-initiated treatment and pharmacist support

**Fig. 1:**
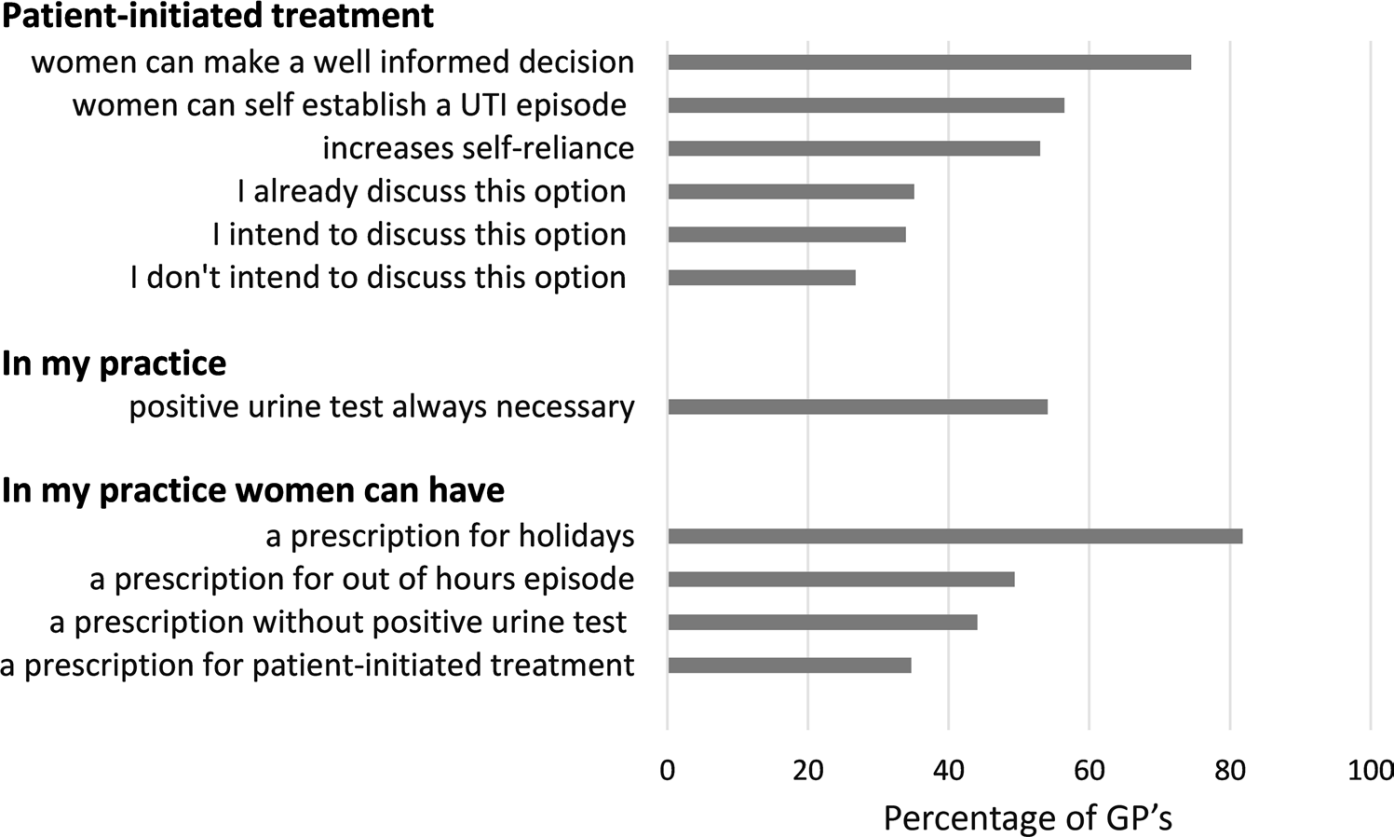
Opinions of general practitioners regarding recurring urinary tract infection (UTI) and patient-initiated treatment

As illustrated in Fig. 1, 56.5% of GPs thought that women could self-diagnose a UTI episode. The majority of GPs (74.5%) agreed that women could self-initiate treatment, provided that the GP discussed the advantages and disadvantages of doing so with their patients. Of the GPs, 35.1% already discussed the possibility of PIT with their patients, whereas 26.8% of GPs had no intention of discussing this. Additionally, 34.7% of the GPs reported that women in their practice could already receive a prescription for PIT if they recognised UTI symptoms. Further, 82.9% of GPs reported that they would provide an antibiotic prescription to take for holidays if a female patient requested it.

As illustrated in Table 2, 32.5% of GPs expected that pharmacists would be willing to check the probability of a recurring UTI episode. Significantly more pharmacists (69.7%) indicated that they would be willing to do this.

**Table 2:** Opinions of general practitioners (GPs) and community pharmacists regarding probability check of an episode of recurring urinary tract infections (UTI) by the community pharmacist

	General practitioners N = 170	Community pharmacists N = 76	*p* Value
Willingness of pharmacists to check the probability of an episode of recurring UTI *			*p* < 0.05
Agree (total)	32.5% (54)	69.7% (53)	
Neutral	38.0% (63)	13.2% (10)	
Not agree (total)	31.9% (53)	17.1% (13)	
Accredited training program for pharmacists is necessary to be able to check the probability of an episode of recurring UTI			
Agree (total)	59.4% (101)	72.4% (55)	
Neutral	22.4% (38)	11.8% (9)	
Not agree (total)	18.2% (31)	3.0% (12)	
Expectation of increase in antibiotics dispensing*			*p* < 0.05
Agree (total)	65.7% (109)	44.7% (34)	
Neutral	24.1% (40)	19.7% (15)	
Not agree (total)	10.2% (17)	35.5% (27)	
Pharmacist expects no objection from the cooperating GPs to pharmacists’ probability check of an episode of recurring UTI, provided agreements are made beforehand			
Agree (total)	n/a	18.4% (14)	
Neutral	n/a	35.5% (27)	
Not agree (total)	n/a	46.1% (35)	
Pharmacists can support patients with recurring UTI withª:			
Information on preventive measures	68.6% (116)**	94.7% (72)**	*p* < 0.05
Information on non-prescription medication	65.7% (111)**	80.3% (61)**	*p* < 0.05
Referral to the GP for prophylaxis	53.3% (96)**	77.6% (59)**	*p* < 0.05
GP conditions for probability check of episode of recurring UTIª:			
Agreements in pharmacotherapeutic audit meeting	67.1% (112)	n/a	
Pharmacist verification of symptoms	49.1% (82)	n/a	
Feedback on dispensing	68.9% (115)	n/a	
Pharmacist check of patient history data	40.7% (68)	n/a	
Preference for organization of probability check in pharmacy*:			*p* < 005
Care provided by pharmacist, with or without support from pharmacy assistant	45.9% (78)	19.7% (15)	
Care provided by pharmacy assistant with protocol after in-service training	30.0% (51)	59.2% (45)	
Care provided by pharmacy assistant with protocol	16.6% (18)	19.7% (15)	
Other	13.5% (23)		

ªTotal > 100%; more than one answer possible

^*^Statistical significance of differences between matrices of agree, neutral and not agree for pharmacists and GPs tested by Chi-square test, difference significant if *p* < 0.05

^**^Statistical significance tested by Chi-square test, difference significant if *p* < 0.05

### Other options for pharmacist support

Additionally, 68.8% of GPs indicated that pharmacists could support women by offering advice on preventive measures to avoid UTI, and 94.7% of the pharmacists thought that they could provide this advice. Some 53.3% of GPs and 77.6% of pharmacists indicated that pharmacists could support women by determining their eligibility for prophylaxis. In the case of prescribing an antibiotic for recurring UTI, 47.1% of the GPs reported that they checked a patient’s eligibility for prophylaxis by consulting the electronic patient record to determine the number of episodes in the previous 12 months.

### Potential consequences of facilitated access to antibiotics with pharmacist support

Of the 170 GPs in the study group, 22.4% expressed concerns about losing information regarding their patients’ antibiotic use, as well as the potential to miss alarming symptoms if women consult their pharmacist instead of the GP for a recurring UTI episode. Moreover, they felt responsible for diagnosing and prescribing. Of the pharmacists, 46.1% expected GPs to object to a probability check of a UTI episode by the pharmacist. If PIT for UTI with pharmacist support were implemented, 65.7% of GPs and 44.7% of community pharmacists expected that there would be an increase in the number of dispensed antibiotics.

### Organization and implementation in pharmacy

According to 59.4% of GPs and 72.4% of community pharmacists, an accredited training program for pharmacists should be mandated before they are allowed to check the probability of an episode of recurring UTI and dispense an antibiotic without a preceding consultation with the GP. In addition, 67.1% of the GPs emphasized the need to make sound agreements about prerequisites in a pharmacotherapeutic audit meeting. The question about the pharmacotherapeutic audit meeting was not in the community pharmacist questionnaire; however, 28.9% of pharmacists spontaneously reported a need for such agreements.

**Fig. 2:**
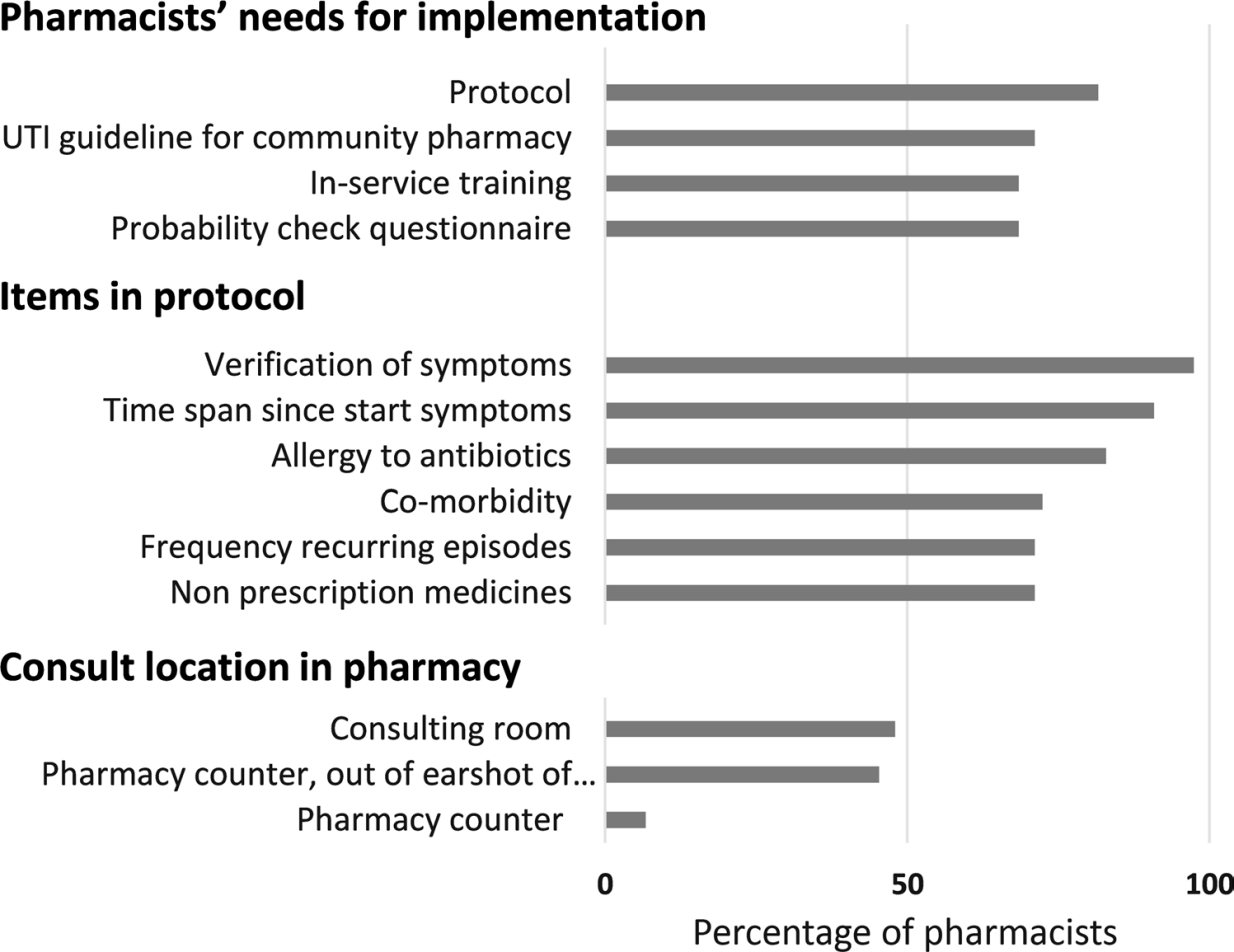
Opinions of community pharmacists regarding pharmacy implementation of probability check of an episode of recurring urinary tract infection (UTI)

As illustrated in Fig. 2, 81.6% of pharmacists reported that they would need a protocol to implement a probability check of an episode of recurring UTI and 68.4% would need a questionnaire to verify patient symptoms. Figure 2 also shows the items pharmacists would prefer in such a protocol. According to 45.9% of GPs and 19.7% of pharmacists, only the pharmacist should be allowed to check the probability of an episode of recurring UTI (see Table 2). Additionally, 93.3% of the pharmacists thought that checking the probability of an episode of recurring UTI should take place out of the earshot of other patients in the pharmacy, and 48.0% of the pharmacists preferred a consulting room (see Fig. 2).

### Gender and age of GPs and pharmacists

No significant gender-based differences were observed in the answers to the reported questions for GPs or community pharmacists. Additionally, no age-based differences were observed for either group based on the categories of < 45 years and ≥ 45 years.

## Discussion

Support of GPs for facilitated access to antibiotic treatment by patient-initiated UTI treatment was limited, even with pharmacist support. The majority of pharmacists was willing to check the probability of an episode of recurring UTI, but both pharmacists and GPs were concerned about overuse of antibiotics. Pharmacist-facilitated access to antibiotics was regarded feasible only if a number of conditions were met.

The majority of GPs thought that women could make a well-informed decision about PIT after discussing advantages and disadvantages with the GP. However, only one-third of the GPs had discussed PIT with their patients. Significantly more community pharmacists expressed willingness to check the probability of an episode of recurring UTI than expected by GPs. Additionally, significantly more GPs than pharmacists expected that the number of antibiotics dispensed would increase if pharmacists were to check the probability of recurring UTI. The most significant concerns mentioned by GPs in the study regarding PIT with pharmacist support were: (1)losing oversight of patients’ antibiotic use, (2)missing potentially alarming symptoms and (3)feeling responsible for diagnosing and prescribing in case of an episode of recurring UTI.

Further, more than half of the GPs emphasized the need to make sound agreements about prerequisites. Pharmacists and general practitioners in the Netherlands have regular pharmacotherapeutic audit meetings, although on varying levels [16]. In practice, the extent of cooperation between pharmacists and GPs also varies. An earlier study found that attempts to encourage one professional group to expand or extend their practice may be perceived as a threat by members of the other group [17]. Clear communication and sufficient time to establish interprofessional trust are potential strategies to mitigate such perceptions [17, 18]. Barriers to effective interprofessional collaboration include perceived hierarchy and power imbalances between the professions and a lack of understanding of each party’s skills and knowledge [19]. A systems thinking approach is supported by a study of a role swap of a pharmacist-led transfer of care [20]. A positive experience of collaboration with a member of the other party led to greater understanding of each group’s capabilities and potential roles. Also, co‐location and other resources to facilitate clear and regular communication between the GP practice and the pharmacy team are important facilitators of interprofessional collaboration [18, 19]. Delegation of care to a pharmacy assistant or technician should also be discussed since significantly more GPs expressed a preference for provision of care by the pharmacist. The education level of pharmacy assistants in practice varies from a basic three year vocational level training up to accredited (self)-care modules on top of this basic training. As of 2004, the pharmacy technician position was added to the pharmacy workforce. To become a pharmacy technician, experienced pharmacy assistants have to complete three years of additional training at the level of higher professional education, which includes theoretical courses and workplace learning [21].

All of these factors must be accounted for prior to implementing a probability check by a pharmacist. Potential GP objections to such practice could be mitigated through pharmacotherapeutic audit meetings that include discussion of these factors, sound agreements about prerequisites for antibiotic dispensing, monitoring and feedback of the number of antibiotic dispensings [8].

Half of the GPs in the study did not support PIT, most likely because they thought confirmation through a diagnostic urine test in the GP practice was necessary for initiation of UTI treatment. Doubts about women’s health literacy and ability to establish whether they might have recurring UTI may contribute to this opinion. A pharmacy study in the Netherlands found that half of pharmacy visitors had limited health literacy skills, with health literacy being defined as the ability to obtain, understand and apply information to make appropriate health decisions [22]. PIT may prove difficult for women who have limited health literacy skills; however, pharmacist support through a probability check and clear communication may facilitate PIT for such women.

Unexpectedly, the majority of GPs in this study reported that they were willing to write a prescription in advance for female patients to take with them when they were going on holiday, though only half thought that women were able to self-diagnose a UTI episode. It is possible that GPs think only women who recognise symptoms will ask for a prescription for holidays. Additionally, women will only ask for such a prescription if they are aware that it is an option.

GPs’ objections to pharmacists checking the probability of a UTI episode may also be related to the fact that antibiotic resistance in the Netherlands is currently lower than in other countries [7], which is likely a consequence of close adherence to GP guidelines. More than 40% of pharmacists expected an increase in antibiotic dispensing. Consequently, prevention of overuse of antibiotics should be an important component of training programs, as well as tools such as guideline and protocol.

More than 70% of pharmacists in the study indicated that they would need a guideline and a training program for implementation. In Canada, a pharmacist UTI guideline was developed that includes symptoms, differential symptoms, co-morbidities and complicating factors [12]. A large prospective registry study indicated that trained pharmacists were able to use the pharmacist UTI guideline to assess and treat UTI in a comparable manner to physicians [10]. Pharmacist management of UTI proved to be highly effective and safe. The clinical cure rate in the Canadian study was 88.9%, and patients reported high satisfaction with this clinical service.

A pharmacist guideline may also be the basis for a protocol. A majority of pharmacists indicated a need for a symptom verification questionnaire as part of a protocol. Prior studies found that a diagnostic algorithm may be a reliable, safe and efficient method to check the probability of an episode of recurring UTI [23, 24].

Nearly 70% of GPs reported that pharmacists could support them by informing women about preventive measures. In GP practice, urine checks by a practice assistant are not usually followed by a GP consultation, and limited information is provided to patients regarding preventive measures.

Half of GPs mentioned that pharmacists could refer female patients to the GP if they seem eligible for prophylaxis. The GP guideline mentions prophylactic treatment and PIT as options for women who have three or more episodes of recurring UTI within a 12-month period [4]. Only half of GPs in the study checked the electronic patient record for the number of episodes in the preceding 12 months, which indicates that screening for viability of prophylactic or PIT is not systematically performed. In the Netherlands, pharmacists can systematically screen their pharmacy information system to determine a patient’s eligibility for prophylaxis and for PIT.

### Strengths and limitations

A strength of the study was that: (1) the practices of GPs and community pharmacists were distributed across the Netherlands, (2) the practices were distributed over rural and urban areas and (3) GPs and pharmacists in the study worked together in a regional care network. Another strength of the study is that the surveys were anonymous; anonymity increases the likelihood that health care providers expressed their true opinions. A limitation of the study is that the number of community pharmacists and GPs was relatively low compared to all community pharmacists and GPs in the Netherlands. Based on a comparison of the percentages of male and female GPs and pharmacists with national data, the group of GPs seems representative contrary to the pharmacists’group [25]. Another limitation is that we failed to ask the community pharmacists about their opinion regarding the conditions for the probability check. Some 70% of pharmacists agreed that an accredited training program is needed to be able to check the probability of an episode of recurring UTI. We did not acknowledge whether this need was for a training program for themselves or for other pharmacists.

## Conclusion

The GPs in this study believed that female patients may recognise recurring UTI; however, they were reluctant to facilitate access to patient-initiated antibiotic treatment of recurring UTI, even with pharmacist support. The majority of pharmacists was willing to check the probability of an episode of recurring UTI. Pharmacist-facilitated access to antibiotics was determined to be a feasible alternative only if a number of conditions were met, including discussion of GP concerns, agreements with GPs, a pharmacist UTI guideline, a questionnaire for verification of symptoms and an accredited training program.
